# An ultrasonographic estimated fetal weight reference for Japanese twin pregnancies

**DOI:** 10.1007/s10396-018-0921-y

**Published:** 2018-12-27

**Authors:** Masaki Sekiguchi, Masashi Mikami, Chie Nakagawa, Mika Ozaki, Shinji Tanigaki, Tohru Kobayashi, Naoyuki Miyasaka, Haruhiko Sago

**Affiliations:** 1 Center for Maternal-Fetal, Neonatal and Reproductive Medicine, National Center for Child Health and Development, 2-10-1 Okura, Setagaya-ku, Tokyo, 157-8535 Japan; 2 Division of Biostatistics, Department of Data Management, Clinical Research Center, National Center for Child Health and Development, Tokyo, Japan; 3 Department of Obstetrics and Gynecology, Kyorin University School of Medicine, Tokyo, Japan; 4 Department of Management and Strategy, Clinical Research Center, National Center for Child Health and Development, Tokyo, Japan; 5 Division of Data Management, Department of Data Management, Clinical Research Center, National Center for Child Health and Development, Tokyo, Japan; 6 Department of Obstetrics and Gynecology, Tokyo Medical and Dental University, Tokyo, Japan

**Keywords:** Estimated fetal weight, Fetal growth, Reference, Twin pregnancy, Ultrasonography

## Abstract

**Purpose:**

The purpose was to establish an estimated fetal weight (EFW) reference for twin pregnancies in Japan and to compare the growth of twins with singletons.

**Methods:**

We retrospectively investigated Japanese women who delivered live-born twins at our center during the period from 2010 to 2016. The main exclusion criteria were monoamniotic twins, fetal reduction, maternal complications, twin–twin transfusion syndrome, fetal congenital anomalies, and patients with their first visit after 16 weeks’ gestation. The EFW was measured longitudinally from 16 to 37 weeks’ gestation. We calculated the posterior predictive distribution using hierarchical Bayesian models and determined the EFW corresponding to each *Z*-score.

**Results:**

A total of 364 women (190 dichorionic and 174 monochorionic) were included, and the total number of examinations was 3952. The EFWs of a *Z*-score of 0 for twins at 20, 28, and 36 weeks’ gestation were 308, 1070, and 2294 g, respectively. The EFW of a *Z*-score of 0 for twins was 98–101% that of singletons until 21 weeks, gradually becoming lower than that of singletons and reaching 90–93% that of singletons after 27 weeks.

**Conclusion:**

We established an EFW reference for Japanese twin pregnancies. The EFW of twins is similar to that of singletons until the mid-second trimester, gradually becoming lower than that of singletons and reaching about 90% that of singletons in the third trimester.

## Introduction

The rate of twin pregnancies has increased, mainly due to maternal aging and fertility treatment over the decades in the USA and Europe [1–3]. In Japan, twin deliveries accounted for about 0.6% of deliveries until the 1980s and then increased to 1.2% in 2004 [4]. The rates of twin deliveries have decreased thanks to recommendations by the Japan Society of Obstetrics and Gynecology about the number of embryos to be transferred [5]; however, the rate remained relatively high at 1.0% in 2016 [6]. Twin pregnancies carry higher risks than singleton pregnancies for maternal and fetal complications, such as preeclampsia, fetal growth restriction, preterm delivery, and perinatal mortality and morbidity [7]. Furthermore, monochorionic (MC) twins carry risks of complications due to discordant blood flow and/or the area of the placenta between fetuses, such as twin–twin transfusion syndrome (TTTS) and selective intrauterine growth restriction [7]. To assess the fetal growth and well-being in twin pregnancies, it is necessary to consider the features of fetal growth in twin pregnancies.

The birthweight of twin pregnancies is reportedly lower than that of singletons [8, 9]; however, the fetal growth of twin pregnancies is usually evaluated using references for singletons in the clinical setting. Although several reports have described references for ultrasonographic estimated fetal weight (EFW) in twin pregnancies [10–14], they had limited clinical use due to the inclusion of various races, small sample sizes, or twins with maternal and/or fetal complications. Furthermore, although birthweights differ by race or ethnicity [15, 16], there have been no reports of EFW references for even Asian twins, let alone Japanese twins specifically.

The purpose of this study was to establish an EFW reference for Japanese uncomplicated twin pregnancies suitable for clinical use, and to compare the growth of twins with that of singletons to clarify the features of growth in twins.

## Materials and methods

We conducted a retrospective cohort study of ultrasonographic EFW in twin pregnancies at the National Center for Child Health and Development in Tokyo, Japan. Japanese women who delivered live-born twins at ≥ 22 weeks of gestation between 2010 and 2016 were included. Pregnancies with oocyte donation, fetal reduction, one or both of the parents not Japanese, maternal complications (e.g., hypertensive disease, diabetes, autoimmune disease), monochorionic monoamniotic twins, fetal death, fetal congenital anomalies, fetal aneuploidy, TTTS, a history of fetal therapy, and women who started to visit our hospital after 16 weeks of gestation were excluded.

The gestational age and chorionicity were confirmed at our hospital or the referring hospital in the first trimester. The gestational age was calculated from the date of ovulation, the date of embryo transfer, last menstrual period, or crown–rump length at 8–10 weeks of gestation, as appropriate. Chorionicity was determined by checking the number of gestational sacs and amnions, “T sign”, or “lambda sign”, i.e., MC diamniotic twins showed two amnions in one gestational sac or “T sign”, and dichorionic (DC) diamniotic twins showed two amnions in two gestational sacs or “lambda sign” [17, 18].

Ultrasound examinations were performed every 4 weeks and every 2 weeks between 16 and 24 weeks of gestation for DC and MC twin pregnancies, respectively, and every 2 weeks between 24 and 36 weeks of gestation and weekly beyond 36 weeks of gestation for both DC and MC twin pregnancies. At each visit, we measured the fetal biparietal diameter, abdominal circumference, and femur length for each fetus according to the standard techniques suggested by the Japan Society of Ultrasonics in Medicine (JSUM) [19]. The EFW was obtained automatically by equipment using the formula proposed by JSUM [19]. Ultrasound examinations were performed by obstetricians and obstetrical ultrasound technologists trained in the Department of Maternal and Fetal Medicine. Ultrasound examinations were performed abdominally using 3- to 5-MHz convex transducers with Voluson E8 (General Electric, Fairfield, USA), Alfa7 and F75 (Hitachi Aloka, Tokyo, Japan), or Aplio XG (TOSHIBA, Tochigi, Japan) ultrasound machines. Women underwent induction of labor or selective cesarean section at 37–38 weeks of gestation unless there were obstetrical indications for delivery, such as spontaneous labor and premature rupture of the membrane.

We applied the normal hierarchical model (hierarchical Bayesian model) to the data. Since the EFW showed linearity by square root transformation, we used the square root of the EFW as an outcome variable. We included the variable of “week” as a fixed effect and the variable of “patient” as a random effect. The prior distribution of the fixed effect was set to follow a normal distribution: the mean comprised the intercept and the slope the “week”, with the variance set to follow the inverse gamma distribution (shape 0.01, scale 0.01). The prior distribution of the random effect was set to follow a bivariate normal distribution, as was the mean (vector ^*t*^[0 0], covariance matrix [*a*1 *a*2], *a*1 = ^*t*^[1000 0], *a*2 = ^*t*^[0 1000]), and the covariance matrix was set to follow the inverse Wishart distribution (degrees of freedom 2, scale parameter matrix [*b*1 *b*2], *b*1 = ^*t*^[0.02 0], *b*2 = ^*t*^[0 20]).

We used the MCMC procedure of the SAS software program, ver. 9.4 (SAS Institute Inc., Cary, USA) to conduct a Markov chain Monte Carlo simulation. The number of Markov chains was 5000, thinning was set at 5, and the number of burn-ins was 1000. After calculating the predictive distribution from the posterior distribution of this model, we squared the value to revert to the original scale. The percentile value was calculated from the squared value of the predictive distribution. The graph shows the values of 2.27, 6.68, 50.00, 93.31, and 97.72% of the predictive distribution, which correspond to *Z*-scores of − 2.0, − 1.5, 0.0, 1.5, and 2.0, respectively.

We compared the predictive EFW reference for twins to the standard reference for singletons used in Japan [19].

## Results

**Fig. 1:**
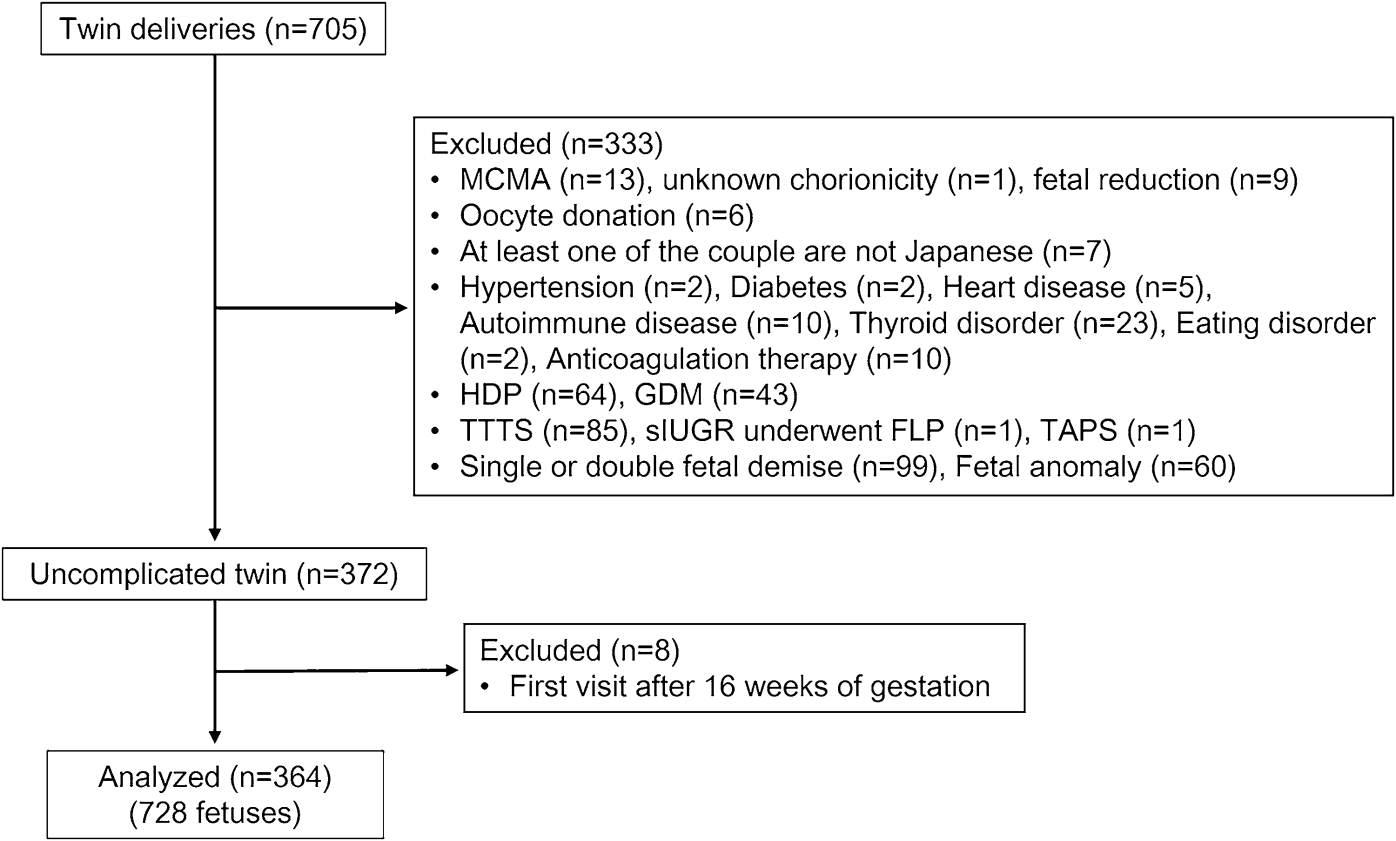
Flowchart of study population selection. *FLP* fetoscopic laser photocoagulation, *GDM* gestational diabetes mellitus, *MCMA* monochorionic monoamniotic, *HDP* hypertensive disorders of pregnancy, *sIUGR* selective intrauterine growth restriction, *TAPS* twin anemia–polycythemia sequence, *TTTS* twin–twin transfusion syndrome

A total of 705 women were recruited for the study and 341 (48.4%) were excluded (Fig. 1). The final study group for the analysis was 364 women (728 fetuses) with a total of 3952 ultrasound examinations yielding 7904 EFW measurements (median of 10.9 times per fetus).

The maternal and neonatal characteristics are shown in Table 1. MC twins accounted for approximately half of the population (47.8%). The median gestational age at delivery was early term of 37.1 weeks, and the median birthweight was 2394 g.

**Table 1:** Maternal and neonatal characteristics

Pregnancy characteristics	Median (interquartile range) or *n* (%)
Maternal age, years	35 (32, 38)
Nulliparous	228 (62.3%)
Smoking during pregnancy	6 (1.7%)
Pregnancy via ART	91 (25.1%)
Prepregnancy BMI, kg/m^2^	19.9 (18.8, 21.6)
GA at delivery, weeks	37.1 (36.7, 37.4)
Monochorionicity	174 (47.8%)
Male fetuses	368 (50.5%)
Birthweight, g	2394 (2162, 2615)

*ART* assisted reproductive technology, *BMI* body mass index, *GA* gestational age

**Fig. 2:**
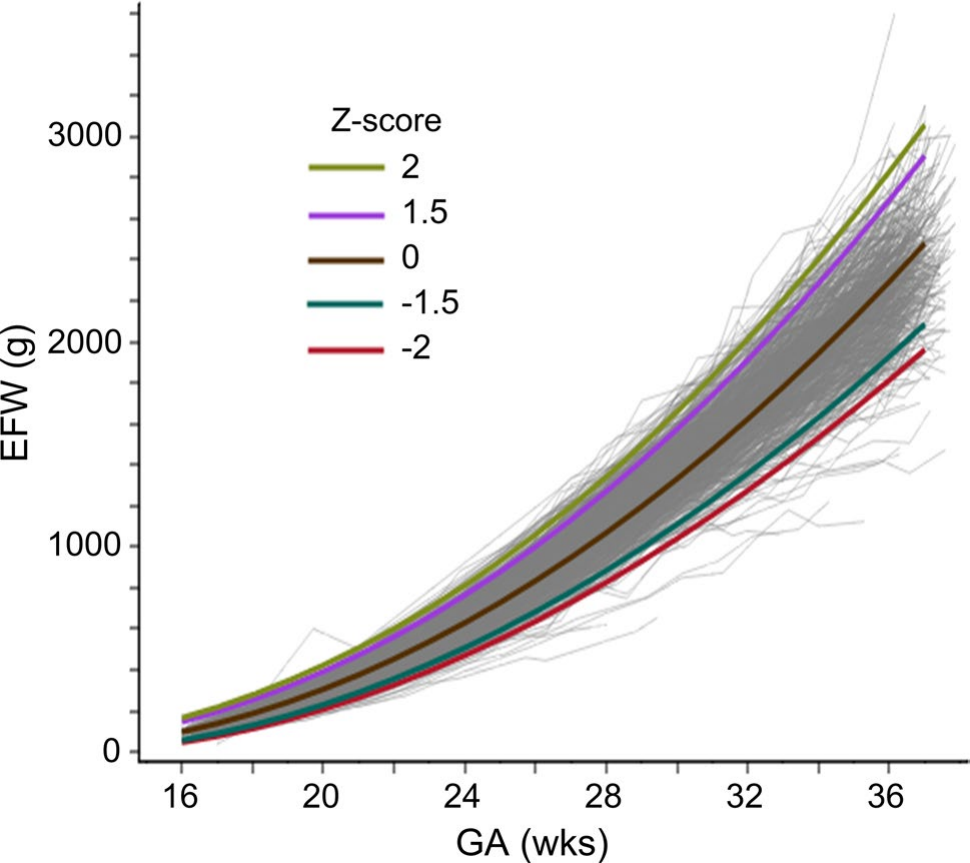
Observed estimated fetal weight (EFW) with predicted EFW reference graphs corresponding to the *Z*-scores of − 2, − 1.5, 0, 1.5, and 2. *EFW* estimated fetal weight, *GA* gestational age

Table 2 shows the EFW corresponding to the *Z*-scores of − 2, − 1.5, 0, 1.5, and 2 at 16–37 weeks of gestation. The weekly gain in EFW increased constantly until term, and the EFW corresponding to the *Z*-score 0 reached 2480 g at 37 0/7 weeks of gestation. The EFWs of the *Z*-score 0 for twins at 20, 28, and 36 weeks were 308, 1070, and 2294 g, respectively. Figure 2 shows the observed EFW with predicted EFW reference graphs corresponding to the *Z*-scores of − 2, − 1.5, 0, 1.5, and 2.

**Table 2:** Reference values of ultrasonographic estimated fetal weight in twins

GA (weeks)	EFW (g) corresponding to each *Z*-score				
	− 2	− 1.5	0	1.5	2
16	48	59	99	149	167
17	79	92	140	198	220
18	115	132	189	256	280
19	159	179	245	321	348
20	209	232	308	394	425
21	266	292	378	474	509
22	328	358	455	564	602
23	397	431	540	661	704
24	472	509	631	766	814
25	552	594	730	880	933
26	639	686	837	1002	1061
27	731	783	950	1133	1198
28	828	886	1070	1272	1343
29	932	995	1198	1420	1498
30	1041	1111	1333	1576	1661
31	1156	1232	1475	1740	1833
32	1276	1360	1625	1913	2015
33	1403	1493	1781	2095	2205
34	1534	1632	1945	2285	2404
35	1671	1777	2116	2484	2613
36	1814	1929	2294	2691	2830
37	1962	2086	2480	2907	3057

*GA* gestational age

**Fig. 3:**
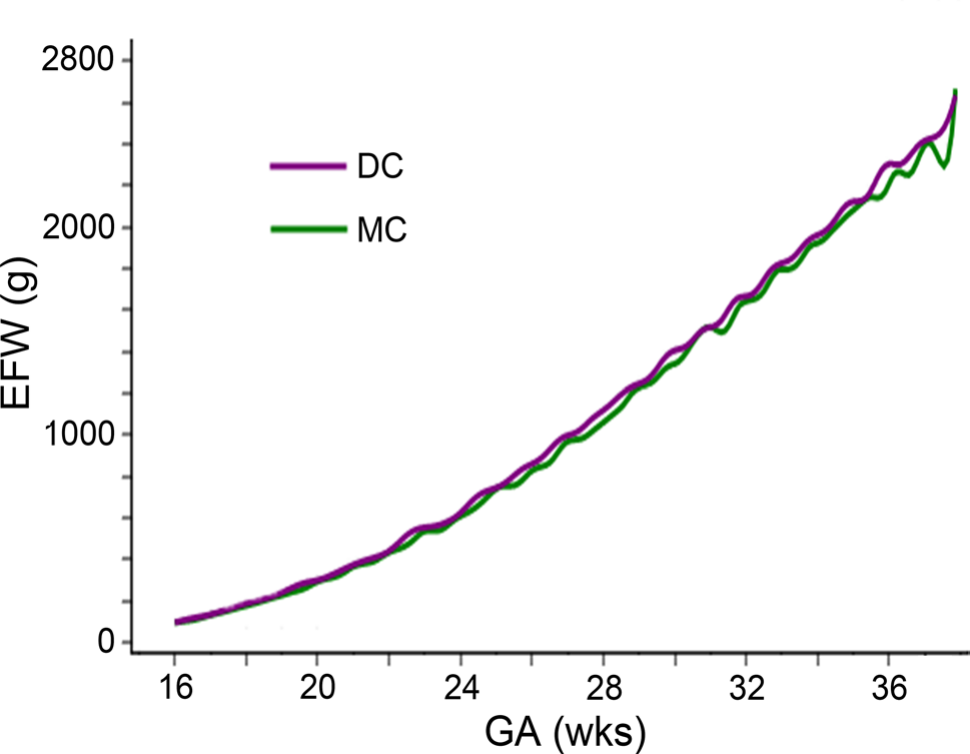
Spline curve of mean estimated fetal weight (EFW) for dichorionic (DC) and monochorionic (MC) twins. MC twins (green line) were slightly lighter than DC twins (purple line); however, the difference was small throughout pregnancy. *EFW* estimated fetal weight, *GA* gestational age

Figure 3 shows the spline curve of the mean EFW for DC and MC twins. MC twins were slightly lighter than DC twins; however, both curves overlapped at many points, and the difference was very small throughout pregnancy.

**Fig. 4:**
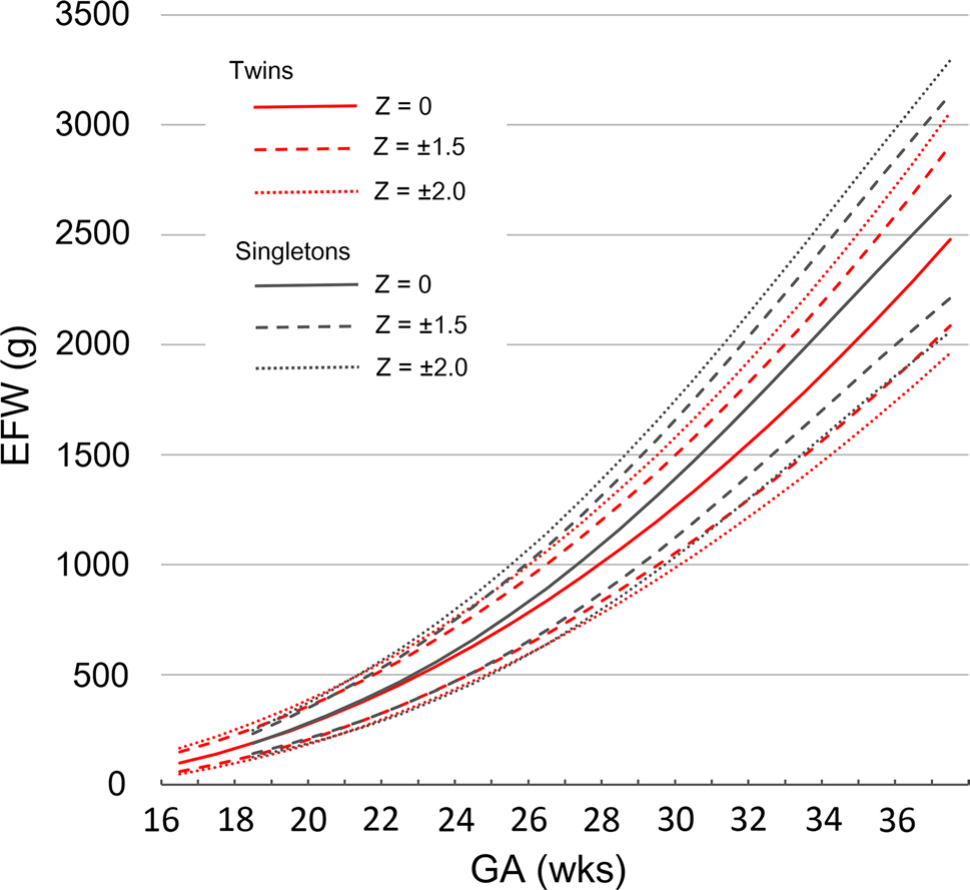
A comparison of the predicted estimated fetal weight reference curve between twins and singletons. The estimated fetal weight (EFW) corresponding to the *Z*-score 0 for twins was almost the same as that for singletons until 21 weeks’ gestation and then gradually became lower than that of singletons, reaching 90–93% that of singletons after 27 weeks. The *Z*-scores of − 1.5 for twins corresponded to − 1.5 for singletons between 20 and 26 weeks and − 2 after 29 weeks. *EFW* estimated fetal weight, *GA* gestational age

Figure 4 shows a comparison of the predicted EFW with *Z*-scores of − 2, − 1.5, 0, 1.5, and 2 between twins and singletons. The EFW corresponding to a *Z*-score of 0 for twins was 98–101% that of singletons until 21 weeks, gradually becoming lower than that of singletons and reaching 90–93% that of singletons after 27 weeks. The *Z*-scores of − 1.5 in the reference for twins corresponded to − 1.5 in the reference for singletons between 20 and 26 weeks and − 2 after 29 weeks.

## Discussion

We established an ultrasonographic EFW reference for Japanese twin pregnancies. To our knowledge, this is the first EFW reference based on a large sample for Asian twin pregnancies.

The EFW at 36 0/7 weeks of gestation was 2294 g, which was similar to that reported by Min et al. [10] and Gabbay-Benziv et al. [14], but lighter than that reported by Shivkumar et al. [13], Araujo Junior et al. [12], and Liao et al. [11] (Table 3). This difference may be mainly due to differences in the study population’s race. The birthweight of Asian infants is reportedly lower than that of white or Hispanic infants [15, 16], and Asian populations have exhibited lower numbers in previous reports (Table 3). Given that birthweights differ among races, it is necessary to use race-matched references for the evaluation of fetal growth.

**Table 3:** Previous reports of estimated fetal weight reference for twin pregnancy

Year	Author	Race	Number of pregnancies and examinations, chorionicity	EFW (g)		
				24 + 0/7 weeks	30 + 0/7 weeks	36 + 0/7 weeks
2000	Min SJ	Black 43% White 35% Hispanic 21% Asian 1%	1831 pregnancies (DC 1282, MC 348)	678 (DC) 666 (MC)	1410 (DC) 1391 (MC)	2359 (DC) 2286 (MC)
2012	Liao AW	Unknown	125 pregnancies (DC 103, MC 16, not determined 6) 807 examinations	621 (DC + MC)	1394 (DC + MC)	2399 (DC + MC)
2014	Araujo JE	Unknown	333 pregnancies (DC176, MC 157) 333 examinations	655 (DC) 631 (MC)	1424 (DC) 1384 (MC)	2530 (DC) 2466 (MC)
2015	Shivkumar S	White 62% Middle East 12% East Asian 10%	642 pregnancies (MC 16%) 3078 examinations	702 (DC) 689 (MC)	1537 (DC) 1479 (MC)	2691 (DC) 2561 (MC)
2017	Gabbay-Benziv R	African-American 55% White 31% Asian 1% Hispanic 1%	2115 pregnancies 5515 examinations (DC 3962, MC 1553)	624 (DC) 601 (MC) 603 (DC + MC)	1434 (DC) 1366 (MC) 1415 (DC + MC)	2308 (DC) 2351 (MC) 2295 (DC + MC)
2018	Sekiguchi M	Asian (Japanese) 100%	364 pregnancies (DC 174, MC 190) 3952 examinations	631 (DC + MC)	1333 (DC + MC)	2294 (DC + MC)

*DC* dichorionic, *EFW* estimated fetal weight, *MC* monochorionic

We feel that our EFW reference is the most suitable for evaluating the fetal growth of Japanese twin pregnancies. Compared to the reference for singletons, the EFW corresponding to a *Z*-score of 0 for twins was 98–101% that of singletons until 21 weeks, gradually becoming lower than that of singletons and reaching 90–93% that of singletons after 27 weeks. Some population-based studies have shown that the birthweight of twins was lower than that of singletons [8, 9], and other studies have shown that the EFW of twin pregnancies was lower than that of singletons [10, 13, 20]. Min et al. [10] reported that the growth of twins became slower than singletons after 30 weeks of gestation (e.g., 1410 g for DC and 1391 g for MC vs. 1359 g for singletons; 2359 g for DC and 2286 g for MC vs. 2813 g for singletons at 30 and 36 weeks, respectively), and Shivkumar et al. [13] reported that the median EFW of DC twins was similar to that of singletons until 32 weeks of gestation (e.g., 1929 g vs. 1953 g at 32 weeks), after which the difference increased (2869 g vs. 3028 g at 37 weeks). However, these reports compared EFW of twins to the singleton chart of Hadlock et al. [21], which was established based on the EFW of different race proportions, i.e., predominantly middle-class whites in the USA. Our results demonstrated differences in the EFW between twins and singletons of the same race from a relatively early gestational age (mid-second trimester). These findings suggest that growth discrepancies between twins and singletons may begin earlier than previously reported.

The *Z*-score of EFW < − 1.5 (equivalent to 6.68th percentile) is used for the diagnosis of fetal growth restriction (FGR) in Japan. *Z*-scores of − 1.5 in the reference for twins corresponded to − 1.5 in the reference for singletons between 20 and 26 weeks and − 2 (equivalent to the 2.27th percentile) after 29 weeks. When diagnosing FGR in twins, it is reasonable to use the reference for singletons until 26 weeks of gestation; however, twins may be overdiagnosed with FGR after 26 weeks when using this reference, especially after 29 weeks of gestation. It might therefore be better to diagnose FGR using the reference for twins in the third trimester.

Several reports have described a relationship between chorionicity and EFW for twins [10, 12–14] (Table 3). Min et al. [10] reported that MC twins were slightly lighter than DC twins; however, the difference was slight, and the proportions of races differed between the DC and MC populations. Araujo et al. [12] also reported that MC twins were slightly lighter than DC twins; however, the number of examinations was small, and the details of the study population were not reported. Shivkumar et al. [13] reported the largest difference between DC and MC twins; however, the MC twins included in the study only accounted for 16% of the population, and the number of examinations at each gestational week was as small as 40. Gabbay-Benziv et al. [14] reported similar EFWs for DC and MC twins based on a large study population; however, the median gestational age at the examination was as late as 35.5 weeks, and the relationship between race proportion and chorionicity was not reported. In contrast, our reference was based on the same race for both DC and MC twins and involved a large number of examinations (more than 100 examinations at each gestational week). The spline curve of the mean EFW showed that MC twins were slightly lighter than DC twins; however, the difference was small throughout pregnancy. This finding may be due to the fact that complicated twins, which are more common in MC twin pregnancies than in DC ones, were excluded in this study. The same reference may be used for DC and MC twins in the clinical setting.

The strengths of our study include the large number of twin pregnancies and examinations. The number of DC and MC twins was approximately the same, and we performed serial ultrasonographic examinations from early in the second trimester to delivery in every pregnancy, making the number of ultrasonographic examinations performed one of the largest ever reported. These characteristics of our population and examinations allowed us to establish optimal references for both DC and MC twins. In addition, our references were based on uncomplicated twin pregnancies, and therefore were suited for a normal reference. Fetal growth is affected by maternal and fetal complications such as hypertensive disease [22], diabetes [22, 23], autoimmune disease [22], and TTTS, and most of the previous reports did not exclude cases with maternal complications [10, 11, 13, 14].

However, several limitations associated with the present study also warrant mention. First, this was a retrospective study conducted at a single center and may have a selection bias. However, because our reference was based on uncomplicated twin pregnancies first encountered before the early second trimester, any EFW error due to the selection bias should not be large enough to hamper clinical use. Second, we compared the reference values for twins not to those of singletons in the same population, but to the reference widely used in Japan. However, the reference in Japan was established in the same race using the same measuring method as our own, and our reference for twins was similar to that of singletons until the mid-second trimester, suggesting it is comparable to the reference for singletons. Finally, whether or not using this reference for twins helps improve the pregnancy outcome in twins is unclear at present. A prospective study of the pregnancy outcome in twins to compare the utility of the references for twins and singletons is needed.

## Conclusion

We established an ultrasonographic EFW reference for Japanese twin pregnancies. The EFW of twins was similar to that of singletons until the mid-second trimester, gradually becoming lower than that of singletons and reaching 90–93% that of singletons in the third trimester. The features of growth in twins were also revealed.
